# Influence of Food Neophobia Level on Fruit and Vegetable Intake and Its Association with Urban Area of Residence and Physical Activity in a Nationwide Case-Control Study of Polish Adolescents

**DOI:** 10.3390/nu10070897

**Published:** 2018-07-13

**Authors:** Dominika Guzek, Dominika Głąbska, Blanka Mellová, Katarzyna Zadka, Katarzyna Żywczyk, Krystyna Gutkowska

**Affiliations:** 1 Department of Organization and Consumption Economics, Faculty of Human Nutrition and Consumer Sciences, Warsaw University of Life Sciences (SGGW-WULS), 159C Nowoursynowska Street, 02-787 Warsaw, Poland; krystyna_gutkowska@sggw.pl; 2 Department of Dietetics, Faculty of Human Nutrition and Consumer Sciences, Warsaw University of Life Sciences (SGGW-WULS), 159C Nowoursynowska Street, 02-787 Warsaw, Poland; dominika_glabska@sggw.pl (D.G.); katarzyna_zadka@sggw.pl (K.Z.); 3 Nutrition, Health and Wellness Unit, Nestlé Polska S.A., 32 Domaniewska Street, 02-672 Warsaw, Poland; blanka.mellova@pl.nestle.com (B.M.); katarzyna.zywczyk@pl.nestle.com (K.Ż.)

**Keywords:** food neophobia, Food Neophobia Scale (FNS), physical activity, urban area, adolescents, fruits, vegetables, #goathletics Study

## Abstract

Among the factors that may influence fruit and vegetable intake, there is a food neophobia level, but the other elements, including physical activity and place of residence, must also be taken into account as interfering ones. The aim of the study was to analyze the association between food neophobia level and the intake of fruits and vegetables in a nationwide case-control study of Polish adolescents (12–13 years), including the influence of gender, the physical activity program participation and the place of residence. The #goathletics Study was conducted among a group of 1014 adolescents, 507 individuals representative for a nationwide physical activity program “Athletics for All” participants (characterized by an active lifestyle) and 507 pair-matched individuals (characterized by sedentary behavior), while 502 were representative for urban and 512 for suburban area. The assessment of food neophobia level was based on the Food Neophobia Scale questionnaire and the assessment of fruit and vegetable intake was based on the validated food frequency questionnaire. It was observed that higher food neophobia level is associated with a lower fruit and vegetable intake, that was stated both for girls and boys, as well as both for individuals characterized by an active lifestyle and those characterized by sedentary behavior, both from urban and suburban area. Food neophobic individuals characterized by an active lifestyle and those from urban areas were characterized by a higher fruit intake than individuals characterized by sedentary behavior and those from suburban areas, from the same food neophobia category. It was found that food neophobia may reduce fruit and vegetable intake, but the physical activity education with peers may reduce the observed influence and should be applied especially in the case of neophobic individuals from suburban areas.

## 1. Introduction

The important health-related behavior that may improve general metabolic health is fruit and vegetable intake. In a number of meta-analyses, it has been observed that fruit and vegetable intake is inversely associated with the risk of coronary heart disease [1,2] and stroke [3,4,5]. The relationship between reduced rates of coronary heart disease and fruit and vegetable intake has been observed to be particularly close in Western countries [6]. The influence of fruit and vegetable intake is observed not only in the risk of developing diseases, but also in mortality rates, as a dose-response association with all-cause mortality, cardiovascular mortality and cancer mortality. This is referred to in the meta-analysis of Wang et al. [7]. Fruit and vegetable intake is especially important in the case of children and adolescents in many cases as it is associated with positive dietary patterns, which should be developed as soon as possible [8]. 

Usually, in order to encourage children to eat more fruits and vegetables, there are a number of obstacles which must be overcome, and among these, one of the most important is food neophobia (defined as reluctance or avoidance of unknown food products) [9]. For children and adolescents, this is one of the most important determinants of food choice that may influence the nutritional value of their diet [10]. In general, it may be stated that food neophobia is inversely associated with the preference for fruits and vegetables, while boys are characterized by a higher level of food neophobia than girls [11]. 

Among the factors that may influence the food neophobia level, as well as the intake of avoided food products, the influence of peers is commonly indicated [12,13], and in the case of adolescents, it is more relevant than the influence of parents [14]. As a result, all the activities practiced with peers, particularly those including eating together, may reduce the food neophobia level and its influence on the diet [13]. 

The other factor that may be associated with food neophobia is sport practicing, as it has been stated that the lack of physical activity is related to cravings for sweets, using food as a reward and for pleasure, that may lead to meal skipping and higher intake of sweets instead of other products [15]. It may result from the fact, that highly rewarding activities (such as sport practicing) are mediated in human brain by the same opioid system, as sweetness palatability [16]. Lack of such activities may lead to urge to increase sweets intake, in order to obtain activation of the system in the other way, that at the same time causes reducing the variety of food products, both being typical for food neophobia [17].

Sport practicing may influence not only the food neophobia level, but also the general quality of diet, as for children and adolescents practicing sport is an additional trigger for following a properly balanced diet and thus obtaining better results [18]. Moreover, the influence of trainers is observed to cause positive changes to eating behaviors of children [19]. Therefore, the influence of both peers and prominent adult social leaders may be supposed to cause changes to eating behaviors of adolescents practicing sports, even if they are food neophobic.

Apart from influence of peers and sport practicing, there are also other factors that may influence in food neophobia level. Among others, there is the residence area, as it is observed that in the case of urban area residents, the food neophobia level is lower, than in the case of rural area ones [20]. Moreover, some authors even hypothesize, that food neophobia may even not exist in the case of urban area residents, however, there is no such evidence in the literature [15].

The aim of the present study was to analyze the association between food neophobia level and the intake of fruits and vegetables in a nationwide case-control study of Polish adolescents aged 12–13 years, including the influence of gender, the physical activity program participation and the place of residence.

## 2. Materials and Methods

### 2.1. Ethics Approval Statement

The #goathletics Study was conducted according to the guidelines laid down in the Declaration of Helsinki, and all the procedures involving human subjects were approved by the Ethics Committee of the Faculty of Human Nutrition and Consumer Sciences of the Warsaw University of Life Sciences (SGGW-WULS) in Warsaw, Poland (No. 16/2017; 19.06.2017).

### 2.2. Study Participants

The study was conducted with two groups of adolescents—a group of individuals participating in a nationwide physical activity program Athletics for All, Lekkoatletyka Dla Każdego (LDK) (individuals characterized by an active lifestyle) and a control group of individuals (characterized by sedentary behavior, not participating in additional sporting activity program after their classes). The LDK program (http://www.lekkoatletykadlakazdego.pl/), which has been conducted in Poland since 2014, is a free-of-charge program for primary school children and adolescents, organized by the Polish Athletic Association and supported by the Ministry of Sport and Tourism and Nestlé Polska S.A. It includes physical activity education and regular athletic training (3 h a week), as well as additional nutritional education. The #goathletics Study was planned to assess the results of the LDK program and to analyze the physical performance, body composition and diet in a group of LDK program participants in comparison with individuals characterized by sedentary behavior.

The study groups were recruited from the category of adolescents aged 12–13 years from all regions of Poland (central, north, north-west, south-west, south, east), while a geographical breakdown was based on Polish statistical data. Both boys and girls were included; however, there was a larger proportion of girls, which was in accordance with the higher number of girls in the LDK program.

The first stage was a purposive sampling of schools and the second stage was a random selection of participants of the study from the chosen schools. The schools in which the LDK program is conducted, and the pair-matched schools in which the LDK program is not conducted (from the same cities) were chosen and the sampling was conducted in such a way as to obtain the assumed geographical breakdown and an equal share of schools from big cities and small towns. 

The inclusion criteria for the group characterized by an active lifestyle were as follows:

- adolescents aged 12–13,
- participating regularly in the LDK program training sessions for at least 1 year,
- not participating in any other physical activity education or nutritional education program,
- written consent agreement of adolescents for participation,
- written consent agreement of the parent/ legal guardian for the participation of their children.

The inclusion criteria for the group characterized by sedentary behavior were as follows:

- adolescents aged 12–13,
- participating in the LDK program neither currently, nor in the past,
- not participating in any other physical activity education or nutritional education program,
- written consent agreement of adolescents for participation,
- written consent agreement of the parent/ legal guardian for the participation of their children.

The exclusion criteria for both groups were as follows:

- missing data in the completed questionnaires,
- diagnosed disabilities in cognitive or motor functions,
- pacemakers and other stimulators,
- diagnosed epilepsy.

**Figure 1:**
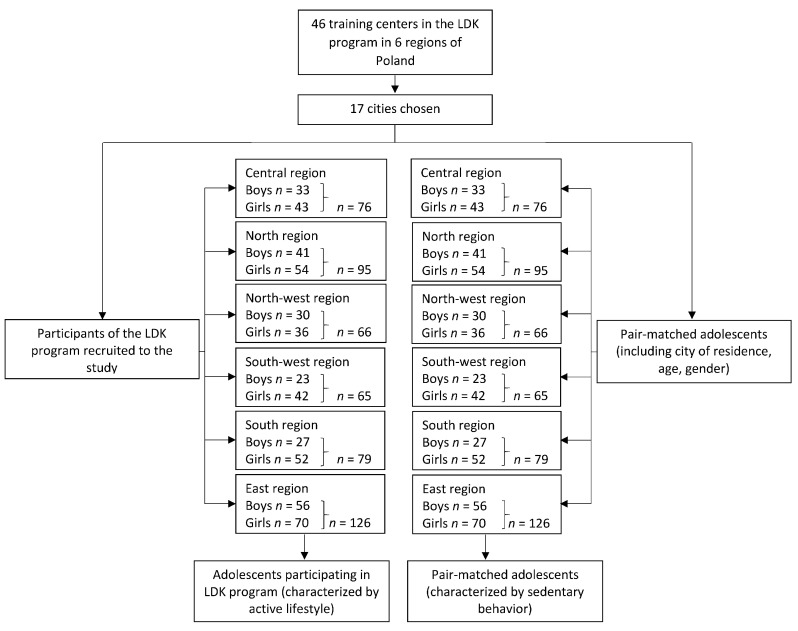
The number of participants of the study from each region of Poland. LDK, Lekkoatletyka Dla Każdego.

The pair-matching of the individuals characterized by sedentary behavior and individuals characterized by an active lifestyle was applied. This included the city of residence, age and gender. As a first stage of the recruitment procedure, individuals characterized by an active lifestyle were randomly recruited from the group of adolescents participating in the LDK program in a specific school. As a second stage of the recruitment procedure, the pair-matched individuals for each individual characterized by an active lifestyle were identified. As a third stage of the recruitment procedure, from the selected pair-matched individuals, for each individual characterized by an active lifestyle, a control individual characterized by sedentary behavior was randomly chosen, while 502 were representative for urban and 512—for suburban areas. The number of participants in the study from each region is presented in Figure 1.

### 2.3. Study Design

The #goathletics Study included an analysis of the physical performance, body composition and diet for a group of LDK program participants in comparison with the group characterized by sedentary behavior. The additional elements included the assessment of the interfering factors influencing the observed associations. Food neophobia was indicated as one of the potential interfering factors; this was based on a previous Polish study conducted in Warsaw [21]. 

The aim of the present analysis was to assess the relationship between the food neophobia level and the intake of fruits and vegetables in the case of boys and girls participating in the LDK program and in the case of the group of boys and girls characterized by sedentary behavior.

#### 2.3.1. Assessment of the Food Neophobia Level

The assessment of the food neophobia level was based on the Food Neophobia Scale (FNS) questionnaire by Pliner and Hobden [22], currently the most widely used instrument to assess food neophobia [9]. Each participant was asked to rate his/her level of agreement with each of the ten sentences, using one of the seven categories of answer (a scale from strongly disagree to strongly agree). For the five “negative item” questions, the scale was reversed during the analysis of data, as is commonly conducted [21,23]. In the present group, Cronbach’s alpha was at a respectable level (0.77; *n* = 1014), which indicated good internal consistency [24].

The calculated food neophobia level ranged from 10 to 70 and for each respondent was attributed to one of the three food neophobia level categories: neophilic (10–30 points), neutral (30–50 points), and neophobic (50–70 points). For neophilic and neophobic individuals, sub-categories were also indicated and, as a result, 5 categories were formulated: neophilic (10–20 points), neophilic tendency (20–30 points), neutral (30–50 points), neophobic tendency (50–60 points) and neophobic individuals (60–70 points). The calculated food neophobia level (number of obtained points) was also analyzed. 

#### 2.3.2. Assessment of the Fruit and Vegetable Intake

To assess the intake of fruits and vegetables, the food frequency questionnaire was applied. This semi-quantified questionnaire was developed for the children and adolescents aged 9–16 and was positively validated using a methodology described previously [25], in a group of 172 children and adolescents. It consisted of 31 questions about the typical intake of food product groups. It had also been employed in our previous study [21] for the assessment of the fruit and vegetable intake in a group of children and adolescents aged 10–12.

In the questionnaire, there were questions about the most important food product groups, while all vegetables were combined as a separate group and all the fruits were also combined as a separate group. Potatoes were categorized as a separate group to avoid misinterpreting them as vegetables. 

For each product item, the typical number of servings consumed per day or week or month (depending on the product) was specified, while a fixed serving size was described for each product. The number of servings was to be expressed in integers and decimal parts. For both fruits and vegetables, the typical serving size indicated in the questionnaire was 100 g (described in the questionnaire also using typical household measures) and respondents were to specify the number of servings consumed per day. 

During the analysis of the data, the declared numbers of servings per day were multiplied by 100 g to obtain the typical intake of fruits/vegetables per day. Fruit and vegetable juices were assessed, but they were not included in the analysis, as a previous study had revealed that the intake of juices is not associated with food neophobia level [21].

### 2.4. Statistical Analysis

The obtained data for fruit and vegetable intake are presented as means ± standard deviation (SD), as well as median, minimum and maximum values. The Shapiro-Wilk test was used to verify the normality of distribution. Cronbach’s alpha coefficient was applied to test the internal reliability of the FNS for analyzed group. The U Mann–Whitney test and the Kruskal–Wallis analysis of variance (ANOVA) test were used to identify the differences between groups due to the non-parametric distributions. The Pearson correlation coefficient (parametric distribution) and Spearman’s rank correlation coefficient (nonparametric distribution) were used to verify the significance of correlations.

The *p* ≤ 0.05 was accepted as a level of significance and *p* ≤ 0.1 as a tendency. Statistical analysis was performed using Statistics software version 8.0 (StatSoft Inc., Tulsa, OK, USA).

## 3. Results

The assessment of vegetable intake for the analyzed groups of boys in various food neophobia categories is presented in Table 1. No differences were observed in vegetable intake between the groups of boys characterized by an active lifestyle and those characterized by sedentary behavior, as well as between the groups of boys from urban and those from suburban area. A significant influence of the food neophobia level in all the groups was noted, as a higher food neophobia level was associated with a lower vegetable intake.

The assessment of vegetable intake for the analyzed groups of girls in various food neophobia categories is presented in Table 2. No differences were observed in vegetable intake between the groups of girls characterized by an active lifestyle and those characterized by sedentary behavior, as well as between the groups of girls from urban and those from suburban area. A significant influence of the food neophobia level in all the groups was noted, as a higher food neophobia level was associated with a lower vegetable intake.

The assessment of fruit intake for the analyzed groups of boys in various food neophobia categories is presented in Table 3. No differences were observed in fruit intake between the groups of boys characterized by an active lifestyle and those characterized by sedentary behavior for the majority of the food neophobia categories. Neophobic boys characterized by an active lifestyle were characterized by higher fruit intake, than those characterized by sedentary behavior, while neophobic tendency boys from urban area were characterized by higher fruit intake, than those from suburban area. At the same time, the significant influence of the food neophobia level in all the groups was noted, as a higher food neophobia level was associated with a lower fruit intake.

The assessment of fruit intake for the analyzed groups of girls in various food neophobia categories is presented in Table 4. No differences were observed in fruit intake between the groups of girls characterized by an active lifestyle and those characterized by sedentary behavior for the majority of the food neophobia categories. Neophobic girls from urban area were characterized by higher fruit intake, than those from suburban area. At the same time, the significant influence of the food neophobia level was noted, as a higher food neophobia level was associated with a lower fruit intake, but not in the group of girls from urban area, as for them the fruit intake did not differ between food neophobia categories.

The analysis of correlations between calculated food neophobia levels and vegetable or fruit intake is presented in Table 5. A negative correlation between the number of FNS points and vegetable or fruit intake was observed for all the groups, and this was stated both for boys and girls.

## 4. Discussion

### 4.1. Influence of Food Neophobia Level on Fruit and Vegetable Intake

Individuals characterized by a higher level of food neophobia are at the same time characterized by a lower willingness to try or eat food products, such as fruits and vegetables, that are unfamiliar compared to other products [26], that may contribute to the lower general diet quality [27]. This may become a serious problem; one consequence is that food neophobia is associated with a lower quality of diet than in the case with neophilic individuals [28].

Our previous study conducted in Warsaw, the capital of Poland, revealed in a group of children and adolescents, aged 10–12, a significant association between the neophobia level and vegetable intake, but not fruit intake [21]. In another Polish study conducted by Kozioł-Kozakowska et al. [28], similar observations were noted in a different age group of preschool children. While comparing mentioned studies, it must be indicated, that for the food neophobia level, the age group may be an important factor, as food neophobic behaviors are reduced with age [29,30]. Taking it into account, the study of Kozioł-Kozakowska et al. [28] must be considered as conducted in a group of children before the cognitive abilities development [31], for whom the high food neophobia level may be typical [32]. At the same time, the previous own study [21], as well as the present one must be considered as conducted in a group of children with developed food behaviors [33] and being in a critical period, as from the age of 13, food behaviors are commonly becoming stable and are transferred to adulthood [34].

### 4.2. Influence of Place of Residence on Association Between Food Neophobia Level and Fruit and Vegetable Intake

When compared with the results of our previous study [21], it must be emphasized that conducting a study in Warsaw, the capital of Poland, may have influenced the observed results. The present study, conducted with adolescents aged 12–13, from all regions of Poland, with an equal number of adolescents from big cities and small towns, revealed, for a total group, associations for both fruits and vegetables. 

The observed differences between the previous study [21] and the present one may have resulted from the different studied populations, as the present study was conducted in a nationwide cohort. The influence of the food neophobia level on fruit and vegetable intake differed between big cities and small towns. In the present study, for the group of girls from urban area, the influence on fruit was not observed, similarly, as was not observed in the previously published study, conducted for the urban area of Warsaw [21].

As it was stated, neophobic adolescents, especially girls, living in big cities may be characterized by a similar fruit intake as neophilic ones, while in small towns they may be characterized by a lower fruit intake. This may be explained as a result of the higher level of access of urban area inhabitants to a variety of food products, which represents exposure to various food product stimuli that may as a consequence reduce the food neophobia level [35]. In the study of Bäckström et al. [36], conducted with a group of adolescents and adults, rural inhabitants were more resistant and suspicious of new types of food products and instead they adhered to natural food, but in general they did not regard eating as a pleasure. 

The new observation from the conducted study is associated with the fact that the correlation observed in the previous study with the urban population [21] was different than that in the present study for a mixed urban-rural population. The difference in the food neophobia level did not result in reduced fruit intake in neophobic female participants in the cities, as was stated in the previous study for the urban population [21]. This may have resulted from the increased possibilities to try various types of fruits, even exotic ones, in the case of the inhabitants of the cities. In the case of the inhabitants of the small towns, due to the lower choice of products, only local products may be consumed.

The indicated results correspond with the general influence of the place of residence on the food neophobia level. In the study of Flight et al. [20], conducted with groups of Australian city students and rural students, the level of food neophobia of students living in rural areas was higher than that of students living in urban areas. This was associated with the fact that city students were also significantly more familiar with various food products and more willing to try unfamiliar products, which resulted from their higher socio-economic status and higher exposure to cultural diversity [20]. Similar observations were indicated in the study of Muhammad et al. [37] as students from rural and semi-rural areas were more neophobic than students from urban ones. Also, in the study of Tuorila et al. [38], conducted with a group of adolescents and adults, food neophobia was inversely associated with the degree of urbanization.

The differences in the food neophobia level between countries must also be noted, as it is well-known that the proportion of neophobic individuals differs between countries. In the study of Ritchey et al. [39], it was observed that individuals from the United States and Finland are characterized by a similar level of food neophobia, but they are more food neophobic than individuals from Sweden. Moreover, in the study of Chung et al. [40], Koreans were characterized by a significantly higher level of food neophobia than individuals from the United States. The observed differences between countries are explained by the association with specific eating habits. Moreover, they may be influenced by the immigration rate, as immigrants try to integrate their eating habits with the host culture or they have bicultural eating habits [41]. As a result, such an attitude also influences the host inhabitants, who are exposed to new food products and dishes associated with immigrant cultures. This may reduce the food neophobia level and increase the willingness to try new products in big cities, as a higher immigration rate is observed in the case of urban areas. 

### 4.3. Influence of Physical Activity With Peers on Association Between Food Neophobia Level and Fruit and Vegetable Intake

In the conducted study, in a group of physically active adolescents, in fact, two factors combined were introduced–the influence of peers and a physical activity itself. Those individuals characterized by an active lifestyle were from the LDK educational program. It included a physical activity education program accompanied by regular athletic training (3 h a week), as well as additional nutritional educational. The nutritional education was not aimed at fruit and vegetable intake, but at general eating habits for training adolescents, so a higher fruit intake was not especially expected in the case of individuals characterized by an active lifestyle. This is associated with the general lack of influence of nutritional education on fruit intake [42], and that even when it is combined with sensory education, it influences the awareness of the sensory aspects of food, but not the food product preferences; this was observed in the review of DeCosta et al. [13]. However, the observed higher intake of fruits may not result from the knowledge that individuals characterized by an active lifestyle obtain during education, but from other factors, such as socialization with peers during trainings, or modeling their eating habits based on the nutritional behaviors of others. 

The social environment inhabited by children and adolescents, which includes their parents, teachers and peers, may influence their eating behavior and remodel it, in both a positive and a negative way, while the food neophobia level may also be reduced by a process of behavior modeling [13]. To be accepted by their peers, adolescents may change their nutritional behaviors and even their nutritional beliefs, to conform with the characteristics of the rest of the group [43]. This is observed especially in the case of adolescents, as parental influence is weaker than in the case of children, and peer influence becomes more important [44]. Similar observations have been reported for the level of physical activity, as the intensity of training increases when the training is accompanied by a peer [45], or a group of peers [46], and peer pressure also increases the level of physical activity of adolescents [47]. 

Most especially in the case of vegetable intake, the influence of peers was proven by Birch [12], as he stated that preschool children, while accompanied by their peers, tend not only to choose non-preferred products if their peers did so, but also to change their food product preferences. The mechanism documented by Birch [12] may mean, that children participating in LDK program (taking part in regular training sessions with their peers and socializing with them) may as a result be characterized by a higher fruit intake than other groups. This corresponds with the observations of Park and Cho [48], as they indicated that the taste education of children aged 7–9 reduces the food neophobia level and increases their willingness to try new food products. Also, Shepard & Dennison [49] indicated that, in the case of adolescents, the influence of peers may be a positive influencing factor that may induce them to increase their fruit and vegetable intake.

Except for the influence of peers, the physical activity itself may influence the food neophobia level, or influence the food product intake, in spite of a food neophobia level (that was observed in the conducted study, as neophobic individuals, in spite of their specified food neophobia, did not reduce fruit intake). Two various mechanisms of the indicated influence may be defined. The first mechanism may be associated with the fact, that spending time on physical activity causes, that adolescents do not have enough time for alternative activities, that in the case of adolescents characterized by sedentary behavior, are often associated with fast food consumption [50]. As a result, the health-promoting choices, that may be offered at home or at school, may be for them easier to obtain and may be more accepted, as indicated by others as promoting better sport results [19].

The second mechanism may be associated with the physiological explanation of action of human brain opioid reward system being associated with the physical activity and sport successes [51]. While the system is being satisfied by the sport, that is practiced, the need to satisfy it by the other stimuli may be decreased, so the need to eat sweets is lower, as it is not needed to improve well-being and relieve stresses [52]. 

### 4.4. Summary

The results observed in a nationwide cohort of Polish adolescents were especially important, as they indicated the association between food neophobia level and fruit intake in this mixed population and the difference between urban and suburban ones. However, it must also be emphasized that rates of urbanization are increasing globally, so the lack of association in the case of fruit intake and urban female teenager population is also important. 

Simultaneously, the results obtained in this nationwide cohort of Polish adolescents enables the highlighting of some differences in fruit intake between adolescents characterized by an active lifestyle and those with sedentary behavior. As a higher intake was stated for individuals characterized by an active lifestyle, the results may indicate a possible way to increase fruit intake through collective physical activity, even if fruit intake education is not conducted.

However, the results of the present analysis lead to the conclusion that further studies into the possibility of reducing food neophobia level through sensory education and socializing are needed. The presented associations allow one to suppose that socializing with peers during sports sessions and eating together may be an activity that encourages people to increase their fruit and vegetable intake, independently of the food neophobia level, and this may be needed to achieve with suburban population. Giving the fact that increasing the fruit and vegetable intake is, for the future health of adolescents, more important than reducing the food neophobia level, sports training programs may be a way to improve not only physical fitness levels, but also fruit and vegetable intake.

Independently of the obtained results, the possible limitations of the study must also be noted. The main limitation is associated with the applied definition of food neophobia level elaborated by Pliner and Hobden [22] and the applied categories of the food neophobia level. As it is uncertain that the definition is applicable to global populations, and there are also other definitions and ways to assess the food neophobia level [9] and novel approaches appear [53], further studies are needed, including alternative ways to assess food neophobia. Moreover, a study should also be conducted on more homogenic groups of individuals, characterized by similar food product availability, culture and family income level.

## 5. Conclusions

In the nationwide cohort of Polish adolescents, aged 12–13, associations were observed between food neophobia level and fruit and vegetable intake, both in groups characterized by an active lifestyle and by sedentary behavior.

The association between food neophobia level and fruit intake may be dependent on the place of residence and it may be not observed for fruit intake in urban girl population.

Socializing with peers during athletics training, may increase fruit intake, independently of the food neophobia level. 

## Figures and Tables

**Table 1:** Vegetable intake (g/day) for boys in Food Neophobia Scale categories—mean ± standard deviation (SD), as well as median, minimum, and maximum values are presented for food neophobia categories and compared between subgroups characterized by an active lifestyle (*n* = 210) and by sedentary behavior (*n* = 210), as well as from urban area (*n* = 210) and suburban area (*n* = 210).

Food Neophobia Category	Neophilic	Neophilic Tendency	Neutral	Neophobic Tendency	Neophobic	*p*-Value
Total (*n* = 420)	204.5 ± 122.4 200 * (50–600)	209.6 ± 161.6 200 * (0–800)	170.0 ± 128.3 100 * (0–800)	100.1 ± 107.3 100 * (0–500)	46.9 ± 71.3 12.5 * (0–200)	0.0000
**Physical activity**						
Boys characterized by an active lifestyle (*n* = 210)	212.5 ± 132.3 200 * (50–600)	231.1 ± 172.5 200 * (0–800)	165.8 ± 109.0 120 * (0–500)	74.1 ± 62.9 50 * (0–200)	108.3 ± 87.8 100 (25–200)	0.0000
Boys characterized by sedentary behavior (*n* = 210)	183.3 ± 98.3 150 * (100–300)	192.4 ± 152.0 125 * (50–800)	173.9 ± 144.3 100 *(0–800)	143.3 ± 148.6 100 * (0–500)	10.0 ± 22.4 40 * (0–50)	0.0030
*p*-Value	0.7681	0.2637	0.6484	0.2037	0.0737
**Place of residence**						
Boys form urban area (*n* = 210)	222.2 ± 83.3 200 * (100–300)	239.5 ± 199.0 200 * (0–800)	171.1 ± 124.0 100 * (0–600)	132.4 ± 132.0 100 *(0–500)	70.0 ± 83.7 50 (0–200)	0.0072
Boys form suburban area (*n* = 210)	192.3 ± 145.6 150* (50–600)	177.5 ± 99.3 175 *(50–500)	168.8 ± 132.9 100* (0–800)	70.8 ± 69.9 50*(0–300)	8.3 ± 14.4 0* (0–25)	0.0000
*p*-Value	0.2426	0.5324	0.7404	0.1399	0.3406	

* distribution different than normal (verified using Shapiro—Wilk test—*p* ≤ 0.05).

**Table 2:** Vegetable intake (g/day) for girls in Food Neophobia Scale categories—mean ± SD, as well as median, minimum, and maximum values are presented for food neophobia categories and compared between subgroups characterized by an active lifestyle (*n* = 297) and by sedentary behavior (*n* = 297), as well as from urban area (*n* = 292) and suburban area (*n* = 302).

Food Neophobia Category	Neophilic	Neophilic Tendency	Neutral	Neophobic Tendency	Neophobic	*p*-Value
Total (*n* = 594)	242.2 ± 151.9 200 * (50–700)	213.4 ± 149.8 200 * (0–800)	179.8 ± 123.8 150 * (0–700)	112.8 ± 90.0 100 * (0–400)	94.5 ± 90.5 75 * (0–300)	0.0000
**Physical activity**						
Girls characterized by an active lifestyle (*n* = 297)	263.9 ± 152.2 275 * (100–700)	222.5 ± 163.1 200 * (0–800)	185.4 ± 125.7 200 * (0–700)	111.5 ± 96.9 100 * (0–400)	108.2 ± 92.4 100 (0–300)	0.0000
Girls characterized by sedentary behavior (*n* = 297)	214.3 ± 152.5 125 * (50–500)	204.3 ± 136.1 200 * (0–600)	174.5 ± 122.1 100 * (0–700)	114.4 ± 85.2 100 * (0–300)	77.8 ± 90.5 50 * (0–300)	0.0007
*p*-Value	0.3141	0.7540	0.3693	0.7915	0.4250	
**Place of residence**						
Girls from urban area (*n* = 292)	223.9 ± 164.4 150 * (50–700)	209.9 ± 154.3 200 * (0–800)	179.3 ± 124.3 150 * (0–700)	137.2 ± 112.6 100 *(0–400)	128.6 ± 122 100 (0–300)	0.0724
Girls from suburban area (*n* = 302)	288.9 ± 108.3 300 (100–400)	217.0 ± 146.3 200 * (0–600)	180.3 ± 123.7 150 * (0–700)	93.8 ± 65.4 100 * (0–300)	76.2 ± 66.9	0.0000
*p*-Value	0.1368	0.6165	0.9303	0.2340	0.4054	

* distribution different than normal (verified using Shapiro—Wilk test—*p* ≤ 0.05).

**Table 3:** Fruit intake (g/day) for boys in Food Neophobia Scale categories—mean ± SD, as well as median, minimum, and maximum values are presented for food neophobia categories and compared between subgroups characterized by an active lifestyle (*n* = 210) and by sedentary behavior *(n* = 210), as well as from urban area (*n* = 210) and suburban area (*n* = 210).

Food Neophobia Category	Neophilic	Neophilic Tendency	Neutral	Neophobic Tendency	Neophobic	*p*-Value
Total (*n* = 594)	243.2 ± 157.4 200 * (33.3–700)	204.0 ± 142.1 200 * (0–600)	189.1 ± 135.0 200 * (0–700)	133.5 ± 113.6 100 * (0–500)	45.8 ± 42.5 0 (0–100)	0.0000
**Physical activity**						
Boys characterized by an active lifestyle (*n* = 210)	276.0 ± 169.5 225 (100–700)	214.5 ± 136.8 200 * (66.7–600)	202.3 ± 137.3 200 * (0–700)	136.0 ± 112.3 100 * (0–500)	88.9 ± 19.2 100 * (66.7–100)	0.0059
Boys characterized by sedentary behavior (*n* = 210)	155.6 ± 72.0 200 * (33.3–200)	195.5 ± 147.2 200 * (0–600)	176.8 ± 132.1 158.3 * (0–700)	129.3 ± 119.7 100 * (0–400)	20.0 ± 27.4 0 * (0–50)	0.0068
*p*-Value	0.1506	0.4201	0.1162	0.5763	0.0369	
**Place of residence**						
Boys from urban area (*n* = 210)	227.8 ± 97.2 200 (100–400)	225.6 ± 153.3 200 * (0–600)	183.6 ± 129.8 200 * (0–700)	173.7 ± 135.8 150 * (0–500)	60.0 ± 41.8 50 (0–100)	0.0170
Boys from suburban area (*n* = 210)	253.8 ± 191.7 200 * (33.3–700)	180.7 ± 126.9 175 * (0–600)	194.6 ± 140.3 200 * (0–700)	97.1 ± 75.3 100 * (0–600)	22.2 ± 38.5 0 * (0–66.7)	0.0007
*p*-Value	0.7894	0.2325	0.6350	0.0451	0.7894	

* distribution different than normal (verified using Shapiro—Wilk test—*p* ≤ 0.05).

**Table 4:** Fruit intake (g/day) for girls in Food Neophobia Scale categories—mean ± SD, as well as median, minimum, and maximum values are presented for food neophobia categories and compared between subgroups characterized by an active lifestyle (*n* = 297) and by sedentary behavior (*n* = 297), as well as from urban area (*n* = 292) and suburban area (*n* = 302).

Food Neophobia Category	Neophilic	Neophilic Tendency	Neutral	Neophobic Tendency	Neophobic	*p*-Value
Total (*n* = 594)	253.1 ± 142.0 200 * (100–800)	220.3 ± 139.3 200 * (0–700)	213.6 ± 136.5 200 * (0–800)	185.7 ± 126.0 200 * (0–500)	137.5 ± 123.4 100 * (0–500)	0.0041
**Physical activity**						
Girls characterized by an active lifestyle (*n* = 297)	277.8 ± 148.7 275 * (100–800)	224.7 ± 137.4 200 * (20–600)	232.0 ± 135.8 200 * (0–700)	179.0 ± 113.9 200 * (0–500)	177.3 ± 150.6 100 * (0–500)	0.0484
Girls characterized by sedentary behavior (*n* = 297)	221.4 ± 131.1 200 * (100–500)	215.9 ± 142.3 200 * (0–700)	196.3 ± 135.3 200 * (0–800)	193.6 ± 141.0 200 * (0–500)	88.9 ± 54.6 100 (0–200)	0.0345
*p*-Value	0.1775	0.6296	0.0022	0.8727	0.2241	
**Place of residence**						
Girls from urban area (*n* = 292)	258.7 ± 158.6 200 * (100–800)	205.2 ± 139.6 200 * (0–700)	200.8 ± 137.4 200 * (0–750)	198.0 ± 145.4 200 (0–500)	214.3 ± 165.1 200 (50–500)	0.3521
Girls from suburban area (*n* = 302)	238.9 ± 92.8 200 (100–400)	236.0 ± 138.5 200 * (30–600)	225.6 ± 135.0 200 * (0–800)	176.0 ± 109.9 200 * (0–500)	96.2 ± 72.1 100 (0–300)	0.0008
*p*-Value	1.0000	0.1566	0.0228	0.6995	0.0372	

* distribution different than normal (verified using Shapiro—Wilk test—*p* ≤ 0.05).

**Table 5:** Analysis of correlations between calculated food neophobia levels and vegetable or fruit intake (*n* = 1014).

Analysed Group	Boys		Girls	
	Vegetables	Fruits	Vegetables	Fruits
**Physical activity**				
Individuals characterized by an active lifestyle	*P* = 0.0000; *R* = −0.3390 *	*P* = 0.0009; *R* = −0.2269 *	*P* = 0.0000; *R* = −0.2679 *	*P* = 0.0061; *R* = −0.1588 *
Individuals characterized by sedentary behavior	*P* = 0.0013; *R* = −0.2201 *	*P* = 0.0031; *R* = −0.2032 *	*P* = 0.0001; *R* = −0.2188 *	*P* = 0.0348; *R* = −0.1225 *
**Place of residence**				
Adolescences from urban area	*P* = 0.0000; *R* = −0.2716 *	*P* = 0.0007; *R* = −0.2324 *	*P* = 0.0016; *R* = −0.1839 *	*P* = 0.0966; *R* = −0.0974 *
Adolescences from suburban area	*P* = 0.0000; *R* = −0.2987 *	*P* = 0.0029; *R* = −0.2040*	*P* = 0.0000; *R* = −0.2985 *	*P* = 0.0007; *R* = −0.1945 *
Total	*P* = 0.0000; *R* = −0.2816 *	*P* = 0.0000; *R* = −0.2168 *	*P* = 0.0000; *R* = −0.2442 *	*P* = 0.0006; *R* = −0.1404 *

* Spearman’s coefficients.
